# Active elderly and health—can moderate exercise improve health and wellbeing in older adults? Protocol for a randomized controlled trial

**DOI:** 10.1186/s13063-021-05278-6

**Published:** 2021-05-07

**Authors:** Mauro Giovanni Carta, Giulia Cossu, Elisa Pintus, Rosanna Zoccheddu, Omar Callia, Giuliana Conti, Mirra Pintus, Cesar Ivan Aviles Gonzalez, Maria Valeria Massidda, Gioia Mura, Claudia Sardu, Paolo Contu, Luigi Minerba, Roberto Demontis, Massimiliano Pau, Gabriele Finco, Eleonora Cocco, Maria Petronilla Penna, Germano Orrù, Goce Kalcev, Federico Cabras, Stefano Lorrai, Andrea Loviselli, Fernanda Velluzzi, Marco Monticone, Enrico Cacace, Mario Musu, Franco Rongioletti, Alberto Cauli, Valeria Ruggiero, Alessandra Scano, Antonio Crisafulli, Sofia Cosentino, Laura Atzori, Elena Massa, Quirico Mela, Dario Fortin, Gianmario Migliaccio, Antonio Egidio Nardi, Matthias Angermeyer, Antonio Preti

**Affiliations:** 1 Department of Medical Sciences and Public Health, University of Cagliari, Cagliari, Italy; 2 Azienda Ospedaliero Universitaria di Cagliari, Cagliari, Italy; 3 Dipartimento di Ingegneria meccanica, chimica e dei materiali, Università degli Studi di Cagliari, Cagliari, Italy; 4 Dipartimento di Pedagogia, psicologia, filosofia, Università degli Studi di Cagliari, Cagliari, Italy; 5 International PhD in Innovation Sciences and Technologies, University of Cagliari, Cagliari, Italy; 6 Vita-Salute - S. Raffaele University, Milan, Italy; 7 Department of Psychology and Cognitive Sciences, University of Trento, Rovereto, Italy; 8 Comitato Olimpico Nazionale Italiano – CONI Sardegna, Cagliari, Italy; 9 Federal University of Rio de Janeiro, Rio de Janeiro, Brazil; 10 Center for Public Mental Health, Gosim, Austria; 11 Department of Neuroscience, University of Turin, Turin, Italy

**Keywords:** Aging, Physical activity, Quality of life, RCT, Cognition, Depression

## Abstract

**Background:**

Aging is marked by a progressive rise in chronic diseases with an impact on social and healthcare costs. Physical activity (PA) may soothe the inconveniences related to chronic diseases, has positive effects on the quality of life and biological rhythms, and can prevent the decline in motor functions and the consequent falls, which are associated with early death and disability in older adults.

**Methods:**

We randomized 120 over-65 males and females into groups of similar size and timing and will give each either moderate physical activity or cultural and recreational activities. Being younger than 65 years, inability to participate in physical activity for any medical reason, and involvement in a massive program of physical exercise are the exclusion criteria. The primary outcome measures are: quality of life, walking speed, and postural sway. Participants are tested at baseline, post-treatment, and 6-month (24 weeks) and 12-month (48 weeks) follow-ups.

**Discussion:**

This study aims at improving the quality of life, wellness, and cognitive functioning in the elderly through a low-cost affordable program of moderate physical activity. Given the growing aging of the world population and the social and economic burden of disability in the elderly, our results might have a major impact on future practices.

**Trial registration:**

ClinicalTrials.gov NCT03858114. Registered on 28 February 2019.

**Supplementary Information:**

The online version contains supplementary material available at 10.1186/s13063-021-05278-6.

## Background

Aging is marked by the rise of chronic diseases with an impact on social and healthcare costs [1]. The European Union has indicated research on active aging as a priority [2].

Physical activity (PA) limits disability related to chronic diseases and has positive effects on the quality of life and biological rhythms [3], and it also prevents the decline in motor functions which helps to prevent falls associated with early death and disability in older adults [4]. PA improves immune responses in old age impacting on metabolic disorders, including diabetes [5]; it lowers cognitive and memory decline [6] and acts in the early stages of depression and in “dysfunctional attitudes” linked to non-acceptance of the limitations imposed by age [7]. Prevention of disability attributable to minor psychological distress can contrast the use of antidepressants, thus sparing side effects and metabolic disorders [4].

To date, most studies on the effectiveness of PA protocols in the elderly were based on trials conducted on small samples, rarely with randomized control designs and still with contradictory indications on timing and optimal method of administration (even though both anaerobic and aerobic activities seem to be useful). While the use of subjective/non-instrumental evaluations in the assessment of motor function outcomes is widespread, the amount of available data from dedicated laboratory equipment—able to provide reliable measures of gait and balance—is limited [8]. Animal studies have shown that PA may influence brain functions through altered blood flow, reduce age-related neuronal loss, and stimulate de-novo synthesis of neurons. These mechanisms might be based on increases in growth factors, neuropeptides, and endorphins [7]. Studies in animals have recently focused on the insulin-like growth factor-1 (IGF-1), which was found upregulated in the brain and the periphery after PA [9]. There is evidence that PA can positively influence the quality of life in the elderly [10], and can improve as well symptoms of depression [11], frequently observed in old people. There is also evidence that acute intense exercise can increase CD4+ and CD8+ T lymphocytes in senescent naïve memory and promote markers of cellular aspects of the immune system in elderly subjects [12]. However, most studies have involved small samples, are based on an acute bout of exercise, and often lack adequate follow-up, and they do not generally provide clarifications as to whether PA can contrast immunosenescence, i.e., the progressive dysfunction of the immune system in the elderly that leads to increased susceptibility to infections and autoimmune disease.

This study set up to investigate (a) whether a moderate activity can be as effective as intense activity in older people. The available studies did not provide an intervention model based on moderate activity, even though this could be the only option viable for the elderly with some medical conditions; (b) the mutual influence of the different variables involved through complex multidisciplinary perspectives (which has been rarely done so far).

We have pioneered the clinical trials and systematic reviews on this topic [3, 13]. The multidisciplinary project we propose is an opportunity to address the above-mentioned issues and to gain further knowledge in the field of active aging. In particular, this study aims at measuring—through a randomized controlled trial—whether an intervention based on moderate PA can improve quality of life (QoL) and motor skills of the elderly living in the community (primary outcomes). The secondary objectives are to measure whether physical activity improves (1) cognitive performance, (2) perception of pain, (3) biological rhythms and immune response linked to metabolic control, (4) community assets (settings, mobility safety, social cohesion), and (5) growth factors and neurotransmitters in the periphery similarly to outcomes in animal studies. The study will also allow investigating the correlation between psychological/motor improvement and the biological markers. The trial will be followed by a 48-week follow-up to investigate whether and to what extent can PA prevent disability and depressive disorders.

## Methods

This is a 12-week randomized controlled trial (RCT) with subsequent 48-week (circa 12 months) follow-up.

### Study design

The study has a longitudinal, two-arm design with participants assigned by randomization to either an active interventional protocol based on physical activity or to a control condition based on cultural and recreational activities focused on education to wellness, cooking, and the history of local culture. Superiority of the intervention group over the control condition is expected in the main outcomes. All participants will be assessed in the pre-treatment period, at the end of the treatment, and at 6 and 12 months after the end of the treatment. The pre-treatment phase consists in a 2-week non-intervention period during which the participants will be assessed about their physical, medical, and psychological status and will receive information about the study so as to get a fully-disclosed informed consent to participate. After inclusion in the study, the participants will receive a 12-week intervention based on either the active condition or the control condition. All interventions will be supervised by staff trained on health issues. After the 12-week intervention, the participants will be assessed to ascertain their physical, medical, and psychological status, and they will be also investigated about potential issues arisen during the intervention and their global satisfaction with the program. The protocol has been prepared according to the Standard Protocol Items: Recommendations for Interventional Trials (SPIRIT) Checklist ([14] see appendix).

### Participants

The target population includes elderly people living at home and recruited through public notices. Age range is as follows: 65 years old and older of both genders. All participants are expected to hold a medical certificate for non-competitive physical activity. Exclusion criteria are as follows: age ≤ 65, BMI > 35, unsuitability for moderate physical activity due to any medical condition, lifetime history of psychosis and/or mania, a certified organic brain disease, and already involved in a program of physical exercise at a level equal or higher than 70% of the expected PA level of the intervention study.

### Measures

The following self-report questionnaires will be used: Short Form Health Survey-12 items (SF-12), as a measure of quality of life (QoL) [15]; Brief Social Rhythms Scale (BSRS), a 10-item measure of the regulation of biological rhythms [16]; International Physical Activity Questionnaire (IPAQ), a 9-item questionnaire aimed at measuring the nature and quantity of physical activity in the past 7 days [17]; and Patient Health Questionnaire-9 items (PHQ-9), a self-administered interview that taps into the nine DSM-IV-TR (Diagnostic and Statistical Manual of Mental Disorders-Fourth Edition; Text Revision) criteria for major depression [18]. The cognitive status of the participants will be measured with the Addenbrooke’s *Cognitive* Examination (ACE), a short cognitive test (about 15 min for administration), which evaluates five cognitive areas: attention/orientation, memory, verbal fluency, language, visual-spatial skills [19]. The Sickness Impact Profile-Roland Scale (SIP-RS) for chronic pain will be used as a measure of chronic pain and its impact on everyday life [20]; the Numeric Pain Rating Scale (NPRS), a visual analog scale on which individuals rate their pain on an eleven-point numerical scale, will be used to obtain an estimate of the current level of pain [21]*.*

### Physiological testing

For all participants, standard anthropometric measures will be acquired: height in meter, weight in kilograms, and waist circumference in centimeters.

Gait analysis, baropodometry, and stabilometry and sit-to-stand (STS) will be used to measure the primary outcomes in relation to PA. Spatiotemporal parameters of gait will be assessed using a validated wireless inertial sensing device. The collected data, transmitted via Bluetooth, will allow obtaining a set of gait parameters: stride length, gait speed, gait cycle duration, stance and swing duration, and double support duration. Balance abilities will be assessed through posture. The following measures will be calculated and analyzed: sway area, center of pressure (CoP) path length, CoP maximum displacement, CoP velocities in anterior–posterior (AP), and medial–lateral (ML) directions [22].

Usual functional mobility will be estimated with the Timed-Up-and-Go (TUG) test, one of the most used measures of mobility, balance, walking ability, and fall risk in older adults. The participant is required to get up from a chair, walk 3 m, go around an obstacle, come back, rotate 180°, and sit down [23]. The TUG summarizes the skills related to walking, equilibrium, coordination, and strength of the lower limbs and is typically assessed in terms of time necessary for its execution. In this study, participants will perform an instrumental version through the use of a wearable inertial sensor applied in the lumbar region at the height of the L2 vertebra. This allows for more detailed data on each of the individual sub-phases.

Laboratory tests will include the following: measurement of glycaemia, total cholesterol, high-density lipoprotein (HDL) *cholesterol*, *triglycerides,* melatonin and insulin-like growth factor-1 (IGF-1) blood levels; complete blood count, erythrocyte *sedimentation rate* (*ESR*); and *C-reactive protein* (*CRP*) levels. As a measure of immune system efficiency, there will be measurements of *immunoglobulin* (Ig) G, A, and M (IgG, IgA, IgM); thymus (T) cells response to mitogens such as phytohemagglutinin (PHA) and cytometric analysis of the T helper (Th) subsets, such as Th1, Th2, and Th17 subsets.

### Procedure and setting

The project will be carried out at the Department of Medical Science and Public Health (coordination actions and data analysis), Laboratory of Movement Analysis at the Cittadella di Monserrato (medical biomechanical and neuropsychological assessments and blood essays), Laboratory of Clinical Biochemistry and Molecular Biology of the Azienda Ospedaliero Universitaria (*AOU*) of Cagliari (blood analysis), at 4 gyms that participated in previous joint studies between the University of Cagliari and CONI (active intervention), and Centro di Riabilitazione of the AOU Cagliari (control condition).

Four test points will be set for each participant: baseline, post-treatment, and 6-month and 12-month follow-ups. Each test point will include the investigation of treatment mechanisms and treatment outcome. Each participant will be assigned to a project coordinator who is responsible for the clinical pathways across the trial. Participants are instructed to report any change in their treatment regimen to the study coordinator they are assigned to. During the trial, all participants will continue their usual treatments and, when they must receive additional prescriptions, they are allowed to take their new treatment unless the treatment implies the loss of eligibility for non-competitive physical activity. In this case, the participant will be withdrawn from the study irrespectively from his/her current allocation. Appropriate debriefing will follow the decision to withdraw the participant from the study.

### Baseline testing

The baseline testing will be in separate days: on 1 day, the participants will be assessed for their physiological and medical condition, also conducting the laboratory tests; on a separate day, the participants will be assessed for their psychological and psychiatric status.

### Randomization

Randomization will be conducted at the University of Cagliari after baseline assessment. Randomization will be computer-based and concealed. The people who will conduct the randomization procedure are blind to the participants’ identities and status and will not receive any information on the participants. Randomization will be by blocks of randomized permutations to experimental treatment with a 1:1 rate; codes will be masked.

### Allocation concealment

Random allocation sequence is generated and distributed in envelopes. The concealment is performed by a member of the research team; this person is not a member of the assessment team nor of the data analysis team. When a person is enrolled, an envelope is opened and the allocation is made known to the person doing the assignment.

### Blinding

The participants will be blind about the condition labeled as active intervention (PA or cultural and recreational activities). The participants will be informed that they will be proposed the participation in activities they can enjoy and which can improve their quality of life as, for example, taking a guided tour of the historical places of the town, receiving a short cooking course, taking part to a low-impact physical activity in a gym, and so on. Information will be given about the pros and cons of each activity, without any detail about which activity could improve their quality of life the most. If someone asks these details, they will be invited to “just try.”

The medical and psychological examiners will be blind to whether the participants have been assigned to the active intervention or the control condition. The project coordinator will schedule meetings with the participants before each appointment, to instruct them not to reveal which treatment they received. In the eventuality that the participants violate this assignment, at the end of the trial, each independent assessor will be asked whether the participant revealed the intervention received; the answer will be recorded to estimate the percentage of blinding violation. The trainers involved in the administration of the active intervention or of the control condition will be blind about the psychological status of the participants. Blinding will be extended to the data analysis team. Data analysts will be blind to treatment allocation since group belonging will be registered as “group A” and “group B,” with no description of the nature of the intervention.

### Pre-treatment period

The pre-treatment phase consists of a 2-week non-intervention period during which the participants will be assessed about their physical, medical, and psychological status and will receive information about the study.

### Treatment

After signing the informed consent, the enrolled subjects will be randomly allocated to experimental treatment by blocks of randomized permutations with a 1:1 rate; codes will be masked. Participants will be evaluated at time zero (t0), baseline; t1, week 12, end of the trial; t2, 24 weeks (circa 6 months) after end of trial; and t3, 48 weeks (circa 12 months) after the end. Blood samples will be conducted at t0 and t1 (3 daily samplings will be made to check the circadian changes). A questionnaire about exercise during the previous six weeks will be done at t0, t2 and t3 (see Table 1 for details; all items can be found in the protocol).

**Table 1:** Schedule of enrolment, interventions, and assessments

	Study period						
Time point	tx	t0		12 weeks	t1	t2	t3
	Enrollment	Baseline	Allocation	Treatment	Post-testing	24 week F-U	48 week F-U
**Enrollment**							
Eligibility screen	X						
Informed consent	X						
Allocation			X				
**Interventions**							
Physical activity				**←**--------→			
Recreational activity				**←**--------→			
Adverse events monitoring				**←**--------→			
**Assessment**							
Medical assessment	X				X		
Anthropometric measurement	X				X		
Demography	X						
**Questionnaires**							
SF-12		X			X	X	X
BSRS		X			X	X	X
IPAQ		X				X	X
PHQ-9		X			X	X	X
SIP-RS		X			X	X	X
NPRS		X			X	X	X
**Cognitive testing**							
ACE		X			X	X	X
**Physiological testing**							
STS		X			X		
TUG		X			X		
**Laboratory tests**							
Glycaemia		X			X		
Total and HDL cholesterol		X			X		
*Triglycerides*		X			X		
Melatonin		X			X		
IGF-1		X			X		
ESR		X			X		
CRP		X			X		
IgG, IgA, IgM		X			X		
PHA test		X			X		
T-helper Th1, Th2, Th17		X			X		

*Abbreviations*: ACE, Addenbrooke’s *Cognitive* Examination; BSRS, Brief Social Rhythms Scale; *CRP, C-reactive protein*; ESR, erythrocyte *sedimentation rate*; *Ig, immunoglobulin*; HDL, high-density lipoprotein *cholesterol*; IGF-1, insulin-like growth factor-1; IPAQ, International Physical Activity Questionnaire; NNT, number needed to treat; *NPRS, Numeric Pain Rating Scale;* PHA, phytohemagglutinin; PHQ-9, Patient Health Questionnaire-9 items; SF-12, Short Form Health Survey 12 items; SIP-RS, Sickness Impact Profile-Roland Scale; STS, sit-to-stand; T, thymus; TUG, Timed-Up-and-Go. All items can be found in the protocol

### Post-treatment

Post-treatment testing is expected to start within 2 weeks after the last treatment session. The post-treatment testing corresponds to the baseline (pre-treatment) testing.

### Follow-up testing at 6 months

The 6-month (24 weeks) testing includes both a medical examination and the psychological testing as in the baseline (pre-treatment) period. However, no laboratory test will be conducted.

### Follow-up testing at 12 months

The 12-month (48 weeks) testing is the same as the 6-month testing.

### Interventions

#### A. Physical exercise

The active intervention will consist in 3 sessions per week. PA was established as 60–84% of the Heart Rate Reserve (HRR); it will monitored continuously during activity and transmitted to the fitness professionals by a telemetry system. The individual HRR for each participant will be assessed according to the Estimated Maximal Heart Rate Formula. Baseline HR will be registered for all participants as a 3-day mean. PA consists of three phases: (1) *warm up,* 10-min, 60% of HRR; (2) *active phase*, 45-min, 60–84% HRR; (3) *cool down*, 10-min, < 60% HRR. The active phase was designed as a mixture of aerobic and anaerobic exercises, integrating drills of “life movements,” strength, and balance.

#### B. Educational and leisure activities

The control group condition will consist in cultural and recreational activities focused on education to wellness, cooking, and the history of local culture headed by an “animator” (with a degree as rehabilitation educator) in groups of the same size (*n* = 15 in each group) and timing as the active intervention group. The control group condition is expected to involve participants for the same amount of time in groups of the same size as the intervention condition, but without the involvement of physical activity, in order to provide a comparator that is devoid of the intended effective stimulus while all the other effects are taken into account (e.g., sociality, amusement, bonding, sharing of time, companionship).

### Primary outcomes

The primary outcomes are quality of life, walking speed, and postural sway. All primary outcomes will be aggregated according to their mean and the outcome will be assessed as a change from the baseline. Table 2 lists the main characteristics of the primary outcomes according to their elements as in Saldanha et al. [24].

**Table 2:** Elements of the completely specified primary outcomes

Domain	Measurement	Metric	Aggregation	Time-point
Quality of life	SF-12	Change from baseline	Mean	At week 12 (end of treatment) At week 48 (end of follow-up)
Walking speed	Gait analysis	Change from baseline	Mean	At week 12 (end of treatment)
Postural sway area	Stabilometry	Change from baseline	Mean	At week 12 (end of treatment)

### Recruitment and eligibility

Participants will be recruited through public notices and referrals by primary health care services. The Italian Olympic Committee (Comitato Olimpico Nazionale Italiano – CONI) will contribute to the study through support in recruiting the elderly (through radio ads, TV, newspapers) and selecting the gyms and the trained staff to carry out the intervention.

To be eligible for the trial, each participant must provide a formal referral from a primary physician to the hospital and a medical certificate of suitability for non-competitive physical activity. After referral by their primary physician, the participant is preliminarily assessed for eligibility. If eligible for inclusion, participants sign an informed consent form and complete self-report baseline measures.

### Strategies to improve adherence

Participants will be randomly inquired about their satisfaction with the activities they are involved in. Reasons for skipping a session will be discussed. After every session, the participants will receive a reminder about the next session by a team member.

### Data management and security

Each potential participant is assigned a unique identification number at the start of the assessment. The study’s coordinators and the research team member in charge of the allocation will know only the pairing between the identity of the participants and their unique identification number. Data are stored according to the hospital’s regional secure rules. Biological material will be stored in a research-specific biobank, according to the procedures of the Regional Committee for Medical and Health Research Ethics. For the analysis, data will be collected in an Excel pre-form. Double-check will be applied routinely to assure that data imputation is correct. All data will be analyzed in an anonymous form and they will be kept for 5 years after the completion of the trial, then they will be destroyed.

### Ethical issues

Each participant will be followed by a project coordinator who is responsible for the clinical pathways across the trial. The project coordinator will be responsible for managing any issue or adverse event that may arise during the trial. Since it is enough to ask the participants what they were doing to identify their experimental/control status, in case of adverse events, no unblinding procedure neither a procedure for revealing the participant’s allocated intervention during the trial has been established. At the end of treatment, each patient will receive clarifications about follow-up testing and will be offered the opportunity to give feedback on treatment and research participation. If any health issue arises during the trial, the project coordinator will refer the participant to the primary physician and, if necessary, will address the participant to the appropriate medical facility for emergency treatment. In case of the emergence of a health condition that precludes further participation in the trial, the participant will be discontinued from trial participation with full disclosure of the reasons. All participants will be insured through a trusted insurance company (name omitted for privacy reasons).

The total load of testing and treatment has been designed to be within the limits that participants aged 65 years and older can tolerate on the basis of past pilot studies done in the same population.

### Adverse event reporting and harms

Physical activity may be a source of concern in the elderly. Adverse events may occur also during leisure activities, e.g., a fall during access to the room where the educational and leisure activities are held. During the trial, and thereafter during the follow-up, adverse events will be continuously collected and assessed at each visit. Adverse events monitoring will be recorded on an ad hoc schedule. Each adverse event will be reported including details about the type of adverse event, its beginning and end (date and time), its severity (from mild to severe), whether or not it could have been expected, its outcome (from completely solved to fatal), and whether or not it was related to the study (certainly/probably/possibly/unlikely/cannot say). Expected adverse events are those that can occur according to the activity (physical or leisure activity), such as muscle strain or fall. Unexpected adverse events are the sudden worsening of a pre-existing minor somatic disorder or cardiovascular events, such as stroke or heart attack, unrelated to the activity.

In case of serious adverse events, a written report will be prepared as per the ethical approval, with details about the participant and the nature of the event. Both the Regional Committee for Medical and Health Research Ethics, region of Sardinia, and the participant’s general practitioner will be notified about any serious adverse event, whether or not it was judged related to the study trial.

Participants incurring serious adverse events from moderate (requiring a relevant change in the treatment) to severe (requiring hospitalization) will be suspended from the study. Mild adverse events (requiring minimal change in the treatment or none at all, or some days of rest at home) will be not a reason for the termination of the trial. Data concerning participants that were suspended from the trial because of adverse events will be entered in the analysis by treatment allocated, as per the CONSORT statement [25].

### Power analysis and statistical analysis

Sample size for expected difference in the primary outcome was based on pilot trials. Effect size of treatment on various outcomes, including motor skills, was 0.48 mean of the standard deviation (SD), with 95% CI = 0.10 to 0.85. As for quality of life and mood, we aimed at detecting clinically significant changes induced by moderate physical exercise. In a recent meta-analysis, moderate-to-vigorous physical exercise was associated with a medium effect size of 0.64 (95% CI, 0.27 to 1.01) in reducing depressive symptoms [26]. Overall, we expect that the experimental intervention could produce a change in the exposed group with an effect size of 0.45 SD. We have determined, by a test of difference between proportions, that a sample of 60 participants per group is required to achieve 80% power to detect a difference with a medium effect size *h* = 0.45, at a two-sided significance level of 0.05. It means that at the estimated power, risk reduction is 0.215, and number needed to treat (NNT) = 5.

The main outcome measures and expected improvement are the following: SF-12 score (10%), walking speed (10%), and postural sway area (20%). Differences in the primary outcomes will be analyzed with a two-tail test for proportions with the Miettinen exact test. A multiple logistic regression will be performed to treat the confounding factors and control the impact of the confounding variables (sex, age, co-morbid condition, previous exercise activity) on outcomes. Survival analyses will be carried out using the Kaplan-Meier curve. Data will be analyzed at the individual level, and clustering (participants having been exposed to the intervention in groups) will be not accounted for in the analysis. Analysis by treatment allocated will be used considering both primary and secondary outcomes. The analysis by treatment allocated will include all participants for which there is data at t0. There will be no interim analyses planned for efficacy or safety since this is a short trial with no administered drug. Analyses will be done with Excel or the Statistical Package for Social Science (SPSS).

### Methods to handle missing data

We do not expect many missing data since the study will be carried out within just one center and the interventional trial has a short duration (12 weeks). However, missing data may occur during the follow-up period. Thus, in the primary analysis, missing data will be replaced by “last-observation-carried-forward.” Then, multiple imputations will be used for sensitivity analyses.

### Oversight and monitoring

The coordinating center staff includes psychiatrists, psychologists, occupational therapists, and nurses, who will be responsible for patient enrollment and the assessment of psychosocial and exercise outcomes; anesthesiologist, cardiologists, internists, dermatologists, orthopedic surgeons, physiatrists, sport medicine physicians, who will be in charge of patient safety and clinical outcome assessment; and geneticists, biologists, laboratory staff, researchers, and data analysts responsible for storing and measuring biomarkers, collecting data, and compiling the case report forms and the database. The coordinating center staff will interlace with the activity of the researchers from the participating center.

The study will have a Steering Committee, a Data Safety Monitoring Committee, and a Quality Control Committee. The Steering Committee comprises the principal investigator, a forensic physician, a dermatologist, an engineer with expertise in gait analysis, an anesthesiologist, a statistician, and a psychiatrist, who also serves as data analyst. The Steering Committee oversees the advancement of the study, identifies problems and difficulties, and implements appropriate corrective measures. The Data Safety Monitoring Committee includes the principal investigator, a nurse, an occupational therapist, an anesthesiologist, and a cardiologist. It is in charge of study monitoring until the end of the follow-up with respect to the study implementation and progress and of making recommendations concerning the continuation, modification, or termination of the trial in relation to unforeseen adverse events of concern. The Quality Control Committee includes the principal investigator, a psychologist, a biologist, and a geneticist and will monitor the study for efficacy and safety. The Data Safety Monitoring Committee and the Quality Control Committee are completely independent from the sponsor that funded the study.

### Dissemination of results

The results of the trial will be disseminated through communications at international meetings and congresses and by publications in high-impact and, preferably, open-access international peer-reviewed journals. Public media will be reached to disseminate the main findings of the trial, and lectures will be given to health care practitioners and voluntary organizations.

## Discussion

Older adults are a rapidly growing group: 22% worldwide and 36% of the Italian population will be over 65 in 2050. Older patients often do not receive adequate and effective help through the current healthcare systems, and comorbid somatic and psychiatric disorders are common in this age class of the population causing an important socioeconomic burden. For these reasons, it is important to provide low-cost, effective treatment for minor psychological disorders that may impact on the somatic health and wellness of elderly. An increase in quality of life may be pivotal in improving the psychological and somatic status of the elderly. To date, the evidence that PA can positively influence the QoL in the elderly [10] and can improve as well the symptoms of depression [11] that are frequently observed in old people, is based on small samples, often without randomized control design and with contradictory indications on timing and optimal modality of administration. The positive impact of PA on the immune system in the elderly is often the result of acute bouts of exercise in studies that often lack adequate follow-up to investigate the persistence of these acute effects [12]. It is still unknown whether the improvement in the quality of life and immunity is a reflection of an impact on biological rhythms; it depends on better wellness and reduced depressive dysfunctional attitudes or still is a direct effect of PA.

This study aims at assessing the effects of moderate PA on an elderly sample’s QoL, biological rhythms (sleep/wake; nutrition and daily life activities), depressive dysfunctional attitudes, static-dynamic balance, cognitive functioning, and at determining the occurrence, if any, of adverse events that are possible in elderly people while performing PA. The study also aims at testing the relationships of such outcomes with the increase of melatonin, IGF-1, and immune response in the blood and to verify the relationships of PA with community assets (settings, mobility safety, social cohesion). The study design includes a control condition that is not expected to provide relevant effects on the main outcomes, although by effect of sociality and the formation of social bonds during the recreational activities, it is still possible that a fraction of the participants may benefit in terms of QoL. The effects of the control condition on the physiological parameters are less likely. In particular, we expect that the PA intervention, but not the control condition focused on recreational activities, will impact on the immune system by improving some of the measures that have been selected to assess its functioning: measurements of IgG, IgA, and IgM; T cells response to mitogens such as PHA and cytometric analysis of the Th1, Th2, and Th17 subsets.

Given the social and economic importance of the issue of disability in the elderly, our results could have a major impact on future practices. The main outcomes of the project are related to the availability of a standardized model of intervention in the real life, while the secondary objectives will provide a set of heuristic hypotheses in a research field defined as a priority for the next Horizon calls. A general objective of the project is also to increase the multi-disciplinary skills and the attitude to joint work in a field that is a primary recipient of EU funding.

The large number of tests and measurements at multiple time points is the main strength of the study, since this allows investigation on the moderators and mediators of treatment efficacy, a point often neglected in past studies. Nevertheless, the number of tests may pose a challenge to the participants, increasing the risk of dropout at post-treatment and follow-up. Many questionnaires also pose the risk of missing data, which is another limitation of the study. Great care will be given to minimize the risk of dropout and incomplete assessment by periodic meetings with the assessors and clarifications to the participants at each test point. It should be noted the lack of accounting for clustering in the analysis, which might bring some potential limitations. However, in this study, the number of clusters is really low, and accounting for clustering, in this case, might lead to biased estimates of standard errors [27].

## Trial status

The study is registered on ClinicalTrials.gov (NCT03858114). The Regional Ethical Committee has approved the study with reference number PG/2018/15546, approved 25 October 2018. The study opened for recruitment in March 2019, and we plan to conclude data collection in June 2020. Protocol version n. 3.2.4; 15 April 2021.

## Supplementary Information

**Additional file 1.** See the SPIRIT 2013 checklist.

## Data Availability

The datasets of this study will be not publicly available due to individual privacy rules.

## Abbreviations

ACE: Addenbrooke’s *Cognitive* Examination
AOU: Azienda Ospedaliero Universitaria
AP: Anterior–posterior
BSRS: Brief Social Rhythms Scale
CONI: *Comitato Olimpico Nazionale Italiano*
CoP: Center of pressure
*CRP*: *C-reactive protein*
DALYs: Disability-adjusted life years
DSM-IV-TR: Diagnostic and Statistical Manual of Mental Disorders-Fourth Edition, Text Revision
ESR: Erythrocyte *sedimentation rate*
HDL: High-density lipoprotein *cholesterol*
HRR: Heart Rate Reserve
*Ig*: *Immunoglobulin*
IGF-1: Insulin-like growth factor-1
IPAQ: International Physical Activity Questionnaire
ML: Medial–lateral
NNT: Number needed to treat
*NPRS*: *Numeric Pain Rating Scale*
PA: Physical activity
PHA: Phytohemagglutinin
PHQ-9: Patient Health Questionnaire-9 items
QoL: Quality of life
RCT: Randomized controlled trial
SD: Standard deviation
SF-12: Short Form Health Survey 12 items
SIP-RS: Sickness Impact Profile-Roland Scale
SPIRIT: Standard Protocol Items: Recommendations for Interventional Trials
SPSS: Statistical Package for Social Science
STS: Sit-to-stand
T: Thymus
t0: Time zero
Th: T-helper
TUG: Timed-Up-and-Go
